# Supplementary material for: “Returning to Ordinary Citizenship”: A Qualitative Study of Chinese PWUD’s Self-Management Strategies and Disengagement Model of Identity
Source: Behav Sci (Basel). 2022 Jul 28;12(8):258. doi: 10.3390/bs12080258 (PMC9404964; doi:10.3390/bs12080258)
Supplement: Supplementary file 1 [file behavsci-12-00258-s001.zip › behavsci-1789915-supplementary.pdf]

Supplementary materials

Interview for PWUDs (original setting)

A Perceptions and reactions to stigma

A1 Do you mind if people around you know your drug use experience?

A2 (If your mind) How can you avoid stigmatization?

A3 Would you like to share some of the discriminatory experiences you have received? (Whether it is in the community or prison)

A4 (If you share), How will you deal with something similar in the future?

B Understanding and narration of drug use

B1 When did you first take drugs? Would you like to share the reasons for taking drugs at that time?

B2 The last time, what was the reason for your relapse?

B3 How do you understand drug use behavior?

B4 Would you like to share experience and story that gave you this cognition?

C The meaning of health

C1 How is your physical condition?

C2 Will you use other substances to replace drug use? (Tobacco, alcohol or others are fine)

C3 (if any) Are you highly dependent on this substance? How often is it used?

C4 Do you think the destructive effects of drug use on the body in the past? Why?

D Understanding of national anti-drug agents

D1 Do you know the community help and education group? What are the main interactions with them?

D2 Do the police usually come to you for random urine tests? Will you feel disturbed?

D3 Would you like to share some methods to avoid being checked by the police or misdiagnosed?

D4 Have you ever had disputes or misunderstandings with the police?

D5 Have you dealt with Narcotics Control Office? Because of what?

D6 Do you know the policies and laws related to drugs?

F Strategies and actions incorporated in life

F1 Where do you currently live? Will the accurate residential address be disclosed to the community teaching team?

F2 How would you rate your current life? Why is this evaluation?

F3 Does your family know about your past drug use experience?

F4 Have you applied for relevant benefits? (For example, subsistence allowances, medical insurance, etc.)

F5 What kind of work are you currently engaged in? Stable or difficult?
